# Examining neurodevelopmental problems in 15q11.2 (BP1‐BP2) copy number variation carriers at ages 9/12 and 18 in a Swedish twin sample

**DOI:** 10.1002/mgg3.2191

**Published:** 2023-05-08

**Authors:** Lina Jonsson, Joanna Martin, Paul Lichtenstein, Patrik K. E. Magnusson, Sebastian Lundström, Lars Westberg, Kristiina Tammimies

**Affiliations:** ^1^ Department of Psychiatry and Neurochemistry Institute of Neuroscience and Physiology at the Sahlgrenska Academy University of Gothenburg Gothenburg Sweden; ^2^ Department of Medical Epidemiology and Biostatistics Karolinska Institutet Stockholm Sweden; ^3^ Centre for Neuropsychiatric Genetics and Genomics Division of Psychological Medicine and Clinical Neurosciences Cardiff University Cardiff UK; ^4^ Gillberg Neuropsychiatry Centre Institute of Neuroscience and Physiology at the Sahlgrenska Academy University of Gothenburg Gothenburg Sweden; ^5^ Department of Pharmacology Institute of Neuroscience and Physiology at the Sahlgrenska Academy University of Gothenburg Gothenburg Sweden; ^6^ Center of Neurodevelopmental Disorders (KIND) Centre for Psychiatry Research Department of Women's and Children's Health Karolinska Institutet and Child and Adolescent Psychiatry Stockholm Health Care Services, Stockholm County Council Stockholm Sweden; ^7^ Astrid Lindgren Children's Hospital Karolinska University Hospital, Region Stockholm Solna Sweden

**Keywords:** 15q11.2, DNA copy number variations, neurodevelopmental disorders

## Abstract

**Background:**

Several copy number variations (CNVs) are associated with increased risk for neurodevelopmental and psychiatric disorders. The CNV 15q11.2 (BP1‐BP2) deletion has been associated with learning difficulties, attention deficit hyperactivity disorder (ADHD), epilepsy, and brain morphology; however, many carriers present mild or no symptoms. Carrying the reciprocal duplication does not seem to confer risk for these disorders or traits. Our aim was to examine the impact of carrying either 15q11.2 deletion and reciprocal duplication on neurodevelopmental problems in a population‐based sample of children.

**Methods:**

Twins with genotype and phenotype information in the Child and Adolescent Twin Study in Sweden (CATSS) were included (*N* = 12,040). We included measures of neurodevelopmental problems (NDPs), including learning problems, from the questionnaire Autism–Tics, ADHD, and other Comorbidities inventory (A‐TAC) at age 9/12, ADHD and autism spectrum disorder (ASD) questionnaires at age 18, as well as information about lifetime psychiatric diagnoses and epileptic seizures. We tested the association between these phenotypic measurements and carrying the 15q11.2 deletion, the reciprocal duplication, and other CNVs with previously reported strong associations with neurodevelopmental and psychiatric disorders (i.e., psychiatric CNVs).

**Results:**

We identified 57 carriers of the 15q11.2 deletion, 75 carriers of the reciprocal duplication, and 67 carriers of other psychiatric CNVs. We did not find an increased risk for NDPs or psychiatric diagnoses in the 15q11.2 deletion carriers. For 15q11.2 duplication carriers, we found an increased risk for math learning problems and fewer self‐reported ADHD symptoms at age 18 but not for other NDPs. In line with previous studies, we found an increased risk of NDPs and other evaluated phenotypes in carriers of psychiatric CNVs.

**Conclusions:**

Our results support previous findings that carrying 15q11.2 deletion does not have a large effect on NDPs in children.

## Graphical abstract

We examined the impact of carrying copy number variations (CNVs) at 15q11.2(DEL/DUP) or other psychiatric CNVs on neurodevelopmental problems in a population‐based cohort of children (N > 12,000). We could not identify a large impact of carrying 15q11.2 DEL; however, we replicate previous findings of more neurodevelopmental problems in psychiatric CNV carriers.

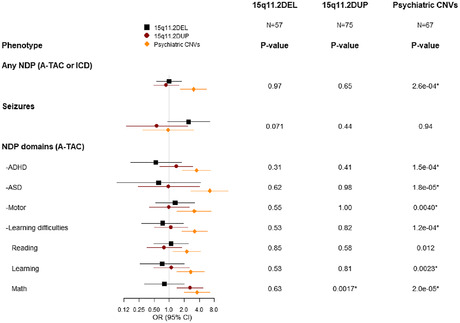

## 1 INTRODUCTION

A major source of genetic variation in the human genome are copy number variations (CNVs), which are structural chromosome rearrangements. There are several CNVs with a strong association with neurodevelopmental disorders such as autism spectrum disorders (ASD) and/or schizophrenia (Gudmundsson et al., 2019; Malhotra & Sebat, 2012; Rees et al., 2016; Stefansson et al., 2014). As these recurrent CNVs are very rare, previous studies have conducted analyses either separately for each CNV or as a group, depending on sample size. One of these CNVs is the rare 15q11.2 deletion (BP1‐BP2, MIM:615656), spanning a non‐imprinted region including four genes (*NIPA1*, *NIPA2*, *CYFIP1*, and *TUBGCP5*). The 15q11.2 deletion (15q11.2DEL) has been robustly associated with susceptibility for neurodevelopmental disorders (Butler, 2017; Gudmundsson et al., 2019) and epilepsy (de Kovel et al., 2010; Lal et al., 2015; Niestroj et al., 2020). In contrast to the 15q11.2DEL, the reciprocal duplication (15q11.2DUP) does not have established pathogenicity for neurodevelopmental disorders and carriers perform on par with the general population on cognitive tests (B. P. Coe et al., 2014; Ulfarsson et al., 2017). However, a 15q11.2 CNV dose‐dependent effect, that is, mirrored effects seen in carriers of the deletion compared to the duplication, on brain white matter microstructure has been shown (Silva et al., 2021; Stefansson et al., 2014).

Interestingly, there is a variability in phenotypic expression in deletion carriers, with a high fraction of carriers displaying no or mild symptoms. The early studies of 15q11.2DEL were mainly based on findings in clinical samples including small control groups showing overestimated effects as the population frequency of the deletion in these control groups were underestimated (Jonch et al., 2019). Currently, the estimated population frequency of the deletion is around 0.3% based on large samples from the general population (Kendall et al., 2017; Stefansson et al., 2014). Population‐based studies, mainly including adults without neurodevelopmental disorders, have shown that carrying the 15q11.2DEL is associated with lower cognitive function (Kendall et al., 2017; Kendall, Bracher‐Smith, et al., 2019; Männik et al., 2015; Williams et al., 2020), dyslexia and dyscalculia (Stefansson et al., 2014; Ulfarsson et al., 2017), and lower educational attainment (Williams et al., 2020). In addition, in brain imaging studies, the 15q11.2DEL has been associated with functional and structural brain alterations (Silva et al., 2021; Ulfarsson et al., 2017; Van der Meer et al., 2020). Taken together, the 15q11.2DEL could be regarded as a pathogenic CNV of mild effect (Jonch et al., 2019).

Our aim was to expand previous findings on the 15q11.2 CNVs in adult populations, by conducting analyses in a large population‐based sample of children. We tested the association between 15q11.2DEL/DUP CNVs and both binary and quantitative measures of neurodevelopmental problems (NDPs) at ages 9/12 and 18 in a Swedish twin sample from the general population. To compare our results for 15q11.2 DEL/DUP to the effects seen in carriers of CNVs known to be strongly associated with psychiatric or neurodevelopmental disorders, we also conducted analyses of all subjects carrying any psychiatric CNV.

## 2 METHODS

### 2.1 Ethical compliance

The study was approved by the regional ethical review board in Stockholm and Karolinska Institutet Ethical Review Board. Parents gave informed consent for study participation on behalf of their children.

### 2.2 Population and measurements

Subjects included in this study are part of the Child and Adolescent Twin Study in Sweden (CATSS), including twins born in Sweden since July 1992 (Anckarsater et al., 2011). The study has been ongoing since 2004 and includes twins at age 9 and initially also age 12, with follow‐up information at age 15, 18, and 24.

NDPs at age 9/12 were based on the Autism–Tics, ADHD, and other Comorbidities inventory (A‐TAC) questionnaire (Hansson et al., 2005; Larson et al., 2010, 2013). The A‐TAC includes questions, with previously validated clinical cut‐offs, in the domains of ASD (17 items, cut‐off ≥4.5), ADHD (19 items, cut‐off ≥6), developmental coordination disorder (DCD)/motor control (1 item, cut‐off ≥0.5), learning difficulties (3 items, cut‐off ≥1) and tics (3 items, cut‐off ≥1.5). All questions are phrased using a lifetime perspective and scored 0 (no), 0.5 (yes, to some extent), and 1 (yes). We included both continuous measures and the low screening cut‐offs for the included NDPs in A‐TAC. Twins included in CATSS were also linked to the Swedish National Patient Registry (NPR), including diagnoses according to the International Statistical Classification of Diseases and Related Health Problems (ICD) versions 9 and 10 for inpatients between 1992 and 2016 and all outpatient visits during 2001 to 2016.

We combined information from A‐TAC and NPR to identify subjects with any NDP. Any NDP was defined as having scores above the low screening cut‐off on any of the A‐TAC domains (ASD, ADHD, DCD/motor control, learning difficulties or tics), or having received an ICD diagnosis for any of the included diagnosed NDPs or anxiety/depression (see Tables S1 and S2).

Based on previous associations between specific learning difficulties and carrying 15q11.2DEL (Stefansson et al., 2014; Ulfarsson et al., 2017), we tested the three specific A‐TAC learning difficulties items about reading, learning, and math difficulties: “Has s/he had more difficulties than expected acquiring reading skills?”, “Is learning slow and laborious?” and “Does s/he have difficulties with basic maths?”. The answers “yes” and “yes, to a certain degree” were coded as having problems and the answer “no” as without any problems.

In addition to information about NDPs from A‐TAC at age 9/12, we included follow‐up measures of specific traits at age 18. We included the full 18 self‐reported questions in the Adult ADHD Self‐Report Scale (ASRS), where the total scores ranged between 0–72, according to the coding by Kessler et al. (2005). For autistic‐like traits at age 18, we included a shorter version of the parent‐rated ASD A‐TAC domain, containing only items based on the DSM‐IV (including 12 questions). To compare twins who participated with those that did not participate at age 18, we only included twins who had turned 18 at time of data extraction (including twins born between 1992 and 1999).

Based on previous associations between 15q11.2DEL and epilepsy, we also included lifetime information about any type of seizures from CATSS at ages 9/12/15 regarding epilepsy and/or fever‐triggered seizures from both questionnaires and interviews as well as lifetime ICD diagnosis (ICD‐10: G40, G41; ICD‐9: 345).

### 2.3 Genotyping and copy number variation (CNV) definitions

DNA collection, genotyping, QC and CNV calling in CATSS has previously been described (Brikell et al., 2018; Martin et al., 2019). We validated the predicted CNV calls for 15q11.2 DEL or DUP CNVs, using the TaqMan Copy Number Assay (Hs04450141_cn) and a reference assay against RNaseP (ref nr: 4403326) from ThermoFisher Scientific (Waltham, Massachusetts, U.S). The TaqMan qPCR validation included DNA from all predicted carriers and their co‐twins. We also included CNVs with previously identified strong associations mainly with ASD and schizophrenia, but these CNVs are also risk factors for other neuropsychiatric disorders (Bradley P. Coe et al., 2014; Gudmundsson et al., 2019; Kendall, Rees, et al., 2019; Malhotra & Sebat, 2012; Marshall et al., 2017; McGrath et al., 2014; Rees et al., 2016; Rees & Kirov, 2021) (Table S2 and Figure S1). Since these psychiatric CNVs individually are rare (<0.5% population frequency), we analyzed carriers of any psychiatric CNVs as a single group. We also used a control group of individuals, with no known recurrent CNVs, their reciprocal DEL/DUP, or CNVs >100 kb in regions that have been associated with genetic syndromes or psychiatric disorders. The final sample size used in the analyses was 12,040 (including 6617 unrelated subjects) who had both phenotype and genotype data.

### 2.4 Statistical analyses

All analyses were performed using logistic or linear generalized estimating equations using the package drgee (Zetterqvist & Sjölander, 2015) in R v. 4.0.4, where family ID was used to cluster the data to account for related twin samples. We used sex and year of birth as covariates in all the statistical analyses. We used Bonferroni correction for multiple testing, giving a corrected *p*‐value <0.0045 (correcting for 11 phenotypes, including 9 binary phenotypes at ages 9/12 and two phenotypes at age 18). Logistic regressions were used for the binary traits of any NDP (A‐TAC or ICD), the low screening cut‐off for A‐TAC domains (ASD/ADHD/motor/learning difficulties), specific learning difficulties items (reading/learning/math), seizures, and for analyses of participation at age 18. Linear regression was used for analyses of quantitative measures of the A‐TAC domains (age 9/12), ASRS (age 18) and ASD symptoms (age 18).

## 3 RESULTS

We identified 57 carriers of 15q11.2DEL, 75 carriers of the reciprocal duplication, 67 carriers of psychiatric CNVs and 11,841 non‐carriers. Table 1 shows the number of monozygotic (MZ) and dizygotic (DZ) twins and twins without their co‐twin for each CNV carrier group. In addition to phenotypes at age 9/12, we analyzed follow‐up information about ADHD and ASD at age 18 for 32 carriers of 15q11.2DEL, 21 carriers of the reciprocal duplication, 30 carriers of psychiatric CNVs, and 5606 controls. The frequency of NDPs within each CNV carrier group at age 9/12 is found in Table S3.

**TABLE 1:** Description of CNV carriers in CATSS.

CNV status	*N*	Mean YOB	Number of twin pairs MZ/DZ/unknown	Number of twins not in pair MZ/DZ/unknown
15q11.2DEL	57	1998	11/5/0	0/25/0
15q11.2DUP	75	1999	13/8/0	0/33/0
Psychiatric CNVs	67	1998	14/16/0	0/27/0
Controls	11,841	1998	2237/3061/1	47/1191/5

Abbreviations: CNV, copy number variation; DEL, deletion; DUP, duplication; DZ, dizygotic; MZ, monozygotic; YOB, year of birth.

**FIGURE 1:**
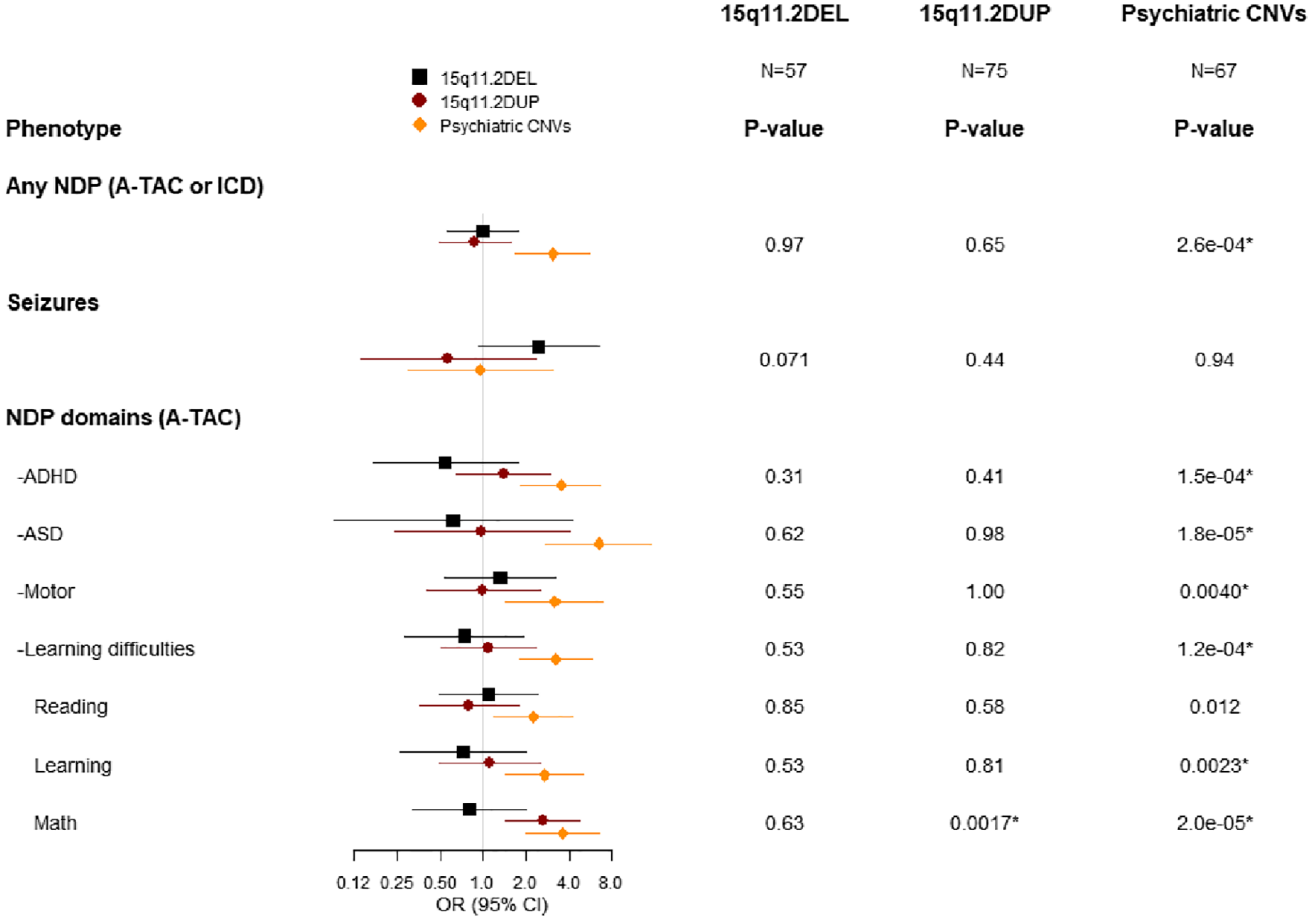
Association results between the binary phenotypes and 15q11.2DEL/DUP as well as psychiatric CNVs compared with controls (*N* = 11,841). CI, confidence interval; OR, odds ratio.

Results from logistic regression analyses of NDPs are shown in Figure 1 and Table S4. We did not find evidence of association between carrying 15q11.2DEL and any NDPs or specific learning difficulties items (continuous trait analyses are shown in Table S4). In 15q11.2DUP carriers, we found an increased risk for math learning problems (OR (95% CI) = 2.6 (1.5–4.7), *p* = 0.0017). The group carrying any psychiatric CNV was associated with more symptoms of NDPs (OR (95% CI) =3.1 (1.7–5.6), *p* = 2.6 × 10^−4^), including a higher risk for ASD symptoms (OR (95% CI) = 6.4 (2.8–15.1), *p* = 1.8 × 10^−5^), ADHD symptoms (OR (95% CI) = 3.5 (1.8–6.7), *p* = 1.5 × 10^−4^), motor problems (OR (95% CI) =3.2 (1.4–7.0), *p* = 0.0040) and learning difficulties (OR (95% CI) = 3.2 (1.8–5.8), *p* = 1.2 × 10^−4^) (Figure 1). For twins with phenotype data also available at age 18, we analyzed continuous measures of ADHD and ASD (Table S4) and found a significant association between carrying psychiatric CNVs and fewer self‐reported ADHD symptoms at age 18 (beta (SE) = −4.56 (1.63), *p* = 0.0053).

We also investigated if there is a difference in NDPs for twins that had participated in the follow‐up at age 18 and those that did not (Table S5). In the non‐carrier control group (*N* = 7288), we discovered more NDPs at age 9/12 in twins not participating in follow‐up at age 18 compared to twins participating (OR (95% CI) = 1.5 (1.3–1.7), *p* = 2.3 × 10^−8^). In the group of psychiatric CNV carriers, 8 of 11 individual twins not participating at age 18 had NDPs at age 9/12 compared to 13 of 30 twins participating at age 18 (OR (95% CI) = 2.4 (0.3–22.7), *p* = 0.44). In 15q11.2DEL carriers, we show that one of six carriers had NDPs at age 9/12 in the non‐participating group, while 6 of 32 twins had NDPs in the participating group (OR (95% CI) = 0.56 (0.1–6.6), *p* = 0.64). Five of 21 carriers of 15q11.2DUP who also participated at age 18 had NDPs compared to five of 15 carriers in the non‐participating group (OR (95% CI) = 3.7 (0.4–32.2), *p* = 0.24). We also compared if the CNV carrier groups were more likely to participate in the follow‐up at age 18 compared to the non‐carrier control group (Table S5). We identified no significant differences on participation in the CNV carrier groups after correction for multiple testing. However, we show a nominal (*p* < 0.05) result indicating that 15q11.2DUP carriers were less likely to participate in the follow‐up at age 18 (OR (95% CI) = 0.4 (0.2–0.9), *p* = 0.035).

## 4 DISCUSSION

Here, we provide additional information on the phenotypic association of 15q11.2DEL/DUP with NDPs, using a longitudinal population‐based child and adolescent twin sample from Sweden. Additionally, we compared the results with the impact of carrying CNVs with a strong association with neurodevelopmental or psychiatric disorders (psychiatric CNVs). Our study did not show an association between a broad definition of any NDPs and carrying the 15q11DEL or the reciprocal duplication. As expected, we saw strong associations with all investigated NDPs for the psychiatric CNV carrier group. Unexpectedly, we found lower scores on the self‐reported ADHD symptoms scale ASRS in carriers participating in the follow‐up at age 18.

Based on the reported increased risk for dyslexia and dyscalculia in 15q11.2DEL carriers (Stefansson et al., 2014; Ulfarsson et al., 2017), we here included measures of learning difficulties capturing problems with reading and math or if learning was slow and laborious. We did not show increased learning problems in 15q11.2DEL carriers, however, carrying 15q11.2DUP associated with increased risk for math learning difficulties similar to the effects seen for the group carrying psychiatric CNVs. As the effects of 15q11.2DUP in large scale studies have not shown clear associations with neurodevelopmental (B. P. Coe et al., 2014) nor learning/math problems (Ulfarsson et al., 2017), this finding needs to be interpreted with caution and evaluated in further studies.

Several studies have shown an increased risk for epilepsy in 15q11.2DEL carriers (de Kovel et al., 2010; Lal et al., 2015; Niestroj et al., 2020). We aimed to broaden these analyses to test if there is an increased risk for febrile seizures or epilepsy in the CNV carriers. However, we could not detect large effects of carrying 15q11.2DEL on risk for seizures in our sample. We identified one concordant twin pair with epilepsy diagnoses and four unrelated twins with a history of fever‐triggered seizures in 15q11.2DEL carriers. Interestingly, we did not find an increased risk for seizures in the group carrying psychiatric CNVs, which includes carriers of several CNVs known to increase the risk for epilepsy (Niestroj et al., 2020).

Although not the main aim of the study, we also demonstrate that individuals with NDPs tend to be less likely to participate in the longitudinal follow‐up in CATSS, which affects the power to do longitudinal phenotype associations for rare CNV groups with higher NDP rates. These selective drop‐outs have also been reported for other longitudinal cohorts and can be associated with erroneous conclusions (Wolke et al., 2009).

The main strength of our study is that we could examine the included CNVs in a well characterized sample of children from the general population, as many previous studies in the general population include only adults. There are also limitations to our study. First, although participation is high in CATSS (80% response rate age 9/12 and 59% response rate at age 18) (Taylor et al., 2019) and results in general can be generalized to the total population, subjects with for example ADHD and learning disabilities were less likely to participate at age 9/12 (Anckarsater et al., 2011). In addition, we show that subjects with NDPs at age 9/12 tend to be less likely to participate at age 18. Taken together with the rare CNVs, we may have limited our possibility to find differences in our sample. We do have the power to detect large effects of carrying the CNVs, as seen for psychiatric CNV carriers, however, we do not have the power to detect small effects in our sample. Finally, even if we used accepted methods for CNV calling and subsequent quality control and qPCR verification, we could still be missing true CNV carriers.

In conclusion, our data supports that 15q11.2DEL can be regarded as a CNV of mild effects (Jonch et al., 2019) and until further evidence of association with phenotypes is available, it is premature for this CNV to be discussed in developmental clinic or prenatal screenings. Our study also provides further evidence for the role of other psychiatric CNVs on continuously distributed NDP symptoms in children in the general population.

## AUTHOR CONTRIBUTIONS

Lina Jonsson, Lars Westberg, and Kristiina Tammimies contributed to the study conception and design. Lina Jonsson, Joanna Martin, and Kristiina Tammimies conducted the CNV calling, CNV verification and statistical analyses. Paul Lichtenstein, Patrik K. E. Magnusson, and Sebastian Lundström managed the data collection in the CATSS study. The first draft of the manuscript was written by Lina Jonsson, Lars Westberg, and Kristiina Tammimies. All the authors read, commented, and approved the final manuscript.

## CONFLICT OF INTEREST STATEMENT

The authors declare no conflict of interest.

## ETHICS STATEMENT

The study was approved by the regional ethical review board in Stockholm and Karolinska Institutet Ethical Review Board.

## Supporting information


Appendix S1.

## Data Availability

Data sharing is not possible due to privacy/ethical restrictions.
