# Supplementary material for: Examining neurodevelopmental problems in 15q11.2 (BP1‐BP2) copy number variation carriers at ages 9/12 and 18 in a Swedish twin sample
Source: Mol Genet Genomic Med. 2023 May 8;11(8):e2191. doi: 10.1002/mgg3.2191 (PMC10422071; doi:10.1002/mgg3.2191)

SUPPORTING INFORMATION

Examining neurodevelopmental problems in 15q11.2(BP1-BP2) deletion carriers at age 9/12 and 18 in a Swedish twin sample

Lina Jonsson, Joanna Martin, Paul Lichtenstein, Patrik Magnusson, Sebastian Lundström, Lars Westberg, Kristiina Tammimies

Supplementary Table 1: Description of included psychiatric ICD diagnoses.

Supplementary Table 2: Description of the CNV carriers.

Supplementary Table 3. Description of the CNV carriers and controls for each of the binary measures of NDPs.

Supplementary Table 4. Association results between continuous measures of neurodevelopmental problems (NDPs) and CNV carriers versus controls (N = 11,841).

Supplementary Table 5. Association results of participation in follow-up at age 18.

Supplementary Figure 1. CNV coordinates in human built hg19 for the included CNVs.

Supplementary Table 1. Description of included psychiatric ICD diagnoses.

Phenotype	Description
ADHD	ADHD/hyperkinetic disorder (ICD10: F90; ICD9:314)
ASD	Autism spectrum disorders (ICD10: F84; ICD9: 299)
Motor Problems	Motor disorders (ICD10: F82; ICD9: 315)
Tic Disorders	Tic disorders (ICD10: F95; ICD9: 307)
Learning difficulties	Intellectual disability (ICD10:F70-79; ICD9:317-319), Language disorders (F80; ICD9:315, 307, 784, V40), scholastic disorders (ICD10: F81; ICD9: 315, V40)
Anxiety	Social anxiety & phobias (ICD10: F40; ICD9: 300), generalized anxiety and panic disorders (ICD10: F41; ICD9: 300), separation anxiety and other childhood-onset anxiety disorders (ICD10: F93; ICD9: 313,309)
Depression	Single and recurrent major depressive disorders (ICD10: F32-F34; ICD9: 296, 298, 300, 301,311)

Supplementary Table 2. Description of the CNV carriers.

CNV	N	ICD diagnoses
**15q11.2(BP1-BP2)**		
DEL	57	ICD10: F32, F41, F81, F90; ICD9: 299
DUP	75	ICD10: F32, F81, F90; ICD9: 317, 319
**Psychiatric CNVs[a]**		
1q21.1 distal_DEL	≤5	-
1q21.1 distal_DUP	≤5	-
2p16.3 (NRXN1) exonic DEL	≤5	-
15q11.2-13.1 DUP	≤5	ICD10:F70,F84,F90; ICD9:299
15q13.3 DEL	≤5	ICD10: F70
16p11.2 distal DEL	≤5	-
16p11.2 proximal DEL	≤5	ICD10:F32,F80
16p11.2 proximal DUP	≤5	-
16p13.11 DEL	≤5	ICD10:F90
16p13.11 DUP	11	ICD10:F32, F70, F84
17p12 DEL	6	ICD10:F32,F33,F90
17p12 DUP	6	ICD10:F41
22q11.2 DEL	≤5	ICD10:F32,F70
22q11.2 DUP	7	ICD10:F70,F71,F82,F84,F90

[a]Additional CNVs where no carriers were found in our sample: 3q29 DEL/DUP, 17q12 DEL/DUP, 7q11.23(WBS) DUP, 7q36.3(VIPR2) DUP and 16p12.1 DEL

Supplementary Table 3. Description of the CNV carriers and controls for each of the included binary measures.

CNV status (N)	Phenotype	N total with problems	N concordant twin pairs with problems	N discordant twins or twins without their co-twin with problems
15q11.2 DEL (N=57)				
	NDP any	16	1	14
	Seizures	6	1	4
	A-TAC: ASD	1	0	1
	A-TAC: ADHD	3	0	3
	A-TAC: Learning	6	1	4
	Item E46: reading	10	2	6
	Item E51: learning	4	0	4
	Item E53: math	5	0	5
	A-TAC: Motor	5	0	5
15q11 DUP (N=75)				
	NDP any	19	2	15
	Seizures	2	0	2
	A-TAC: ASD	2	0	2
	A-TAC: ADHD	9	1	7
	A-TAC: Learning	11	2	7
	Item E46: reading	10	2	6
	Item E51: learning	8	1	6
	Item E53: math	18	3	12
	A-TAC: Motor	5	0	5
Psychiatric CNVs (N=67)				
	NDP any	37	10	17
	Seizures	3	0	3
	A-TAC: ASD	11	4	3
	A-TAC: ADHD	18	4	10
	A-TAC: Learning	23	5	13
	Item E46: reading	21	5	11
	Item E51: learning	15	2	11
	Item E53: math	20	4	12
	A-TAC: Motor	13	4	5
Controls (N=11,841)				
	NDP any	3280	737	1806
	Seizures	536	61	414
	A-TAC: ASD	315	31	253
	A-TAC: ADHD	1048	128	792
	A-TAC: Learning	556	0	556
	Item E46: reading	774	0	774
	Item E51: learning	1128	133	862
	Item E53: reading	1290	202	886
	A-TAC: Motor	778	72	634

Supplementary Table 4. Association results between continuous measures of neurodevelopmental problems (NDPs) and CNV carriers versus controls (N = 11,841).

	15q11.2DEL $N_{age9/12}$ = 57 N_{age18} = 32		15q11.2DUP $N_{age9/12}$ = 75 N_{age18} = 21		Psychiatric CNVs[a] $N_{age9/12}$ = 67 N_{age18} = 30	
**Phenotype**	**beta (se)**	**P-value**	**beta (se)**	**P-value**	**beta (se)**	**P-value**
NDPs[b] (age 9/12)	-0.086 (0.46)	0.85	0.58 (0.62)	0.35	3.55 (1.19)	**0.0029**
ASD	-0.12 (0.16)	0.46	0.024 (0.15)	0.87	1.22 (0.48)	0.012
ADHD	0.020 (0.36)	0.96	0.44 (0.42)	0.29	1.85 (0.64)	**0.0035**
Learning difficulties	-0.044 (0.063)	0.48	0.11 (0.11)	0.32	0.36 (0.12)	**0.0020**
Motor control	<0.01	0.83	<0.01	0.96	0.085 (0.041)	0.038
Tics	0.049 (0.056)	0.38	<0.01	0.94	0.024 (0.060)	0.69
ASD (age 18)	-0.06 (0.20)	0.78	-0.22 (0.15)	0.13	0.67 (0.40)	0.093
ASRS (age 18)	-1.20 (1.51)	0.43	-0.62 (2.43)	0.80	-4.56 (1.63)	**0.0053**

[a]Included psychiatric CNVs are found in supplementary table 2. [b]NDPs at age 9/12 based on A-TAC.

Supplementary Table 5. Association results between participation in follow-up at age 18.

	15q11.2DEL $N_{follow-up}$ = 32 $N_{not\_follow-up}$ = 6		15q11.2DUP $N_{follow-up}$ = 21 $N_{not\_follow-up}$ = 15		Psychiatric CNVs[a] $N_{follow-up}$ = 30 $N_{not\_follow-up}$ = 11		Controls $N_{follow-up}$ = 5,606 $N_{not\_follow-up}$ = 1,682	
**Phenotype**	**OR (95% CI)**	**P-value**	**OR (95% CI)**	**P-value**	**OR (95% CI)**	**P-value**	**OR (95% CI)**	**P-value**
Participation follow-up[b]	1.7 (0.6-5.1)	0.36	0.4 (0.2-0.9)	0.035	0.9 (0.4-1.9)	0.73	-	-
NDPs at age 9/12[c]	0.56 (0.1-6.6)	0.64	3.7 (0.4-32.2)	0.24	2.4 (0.3-22.7)	0.44	1.5 (1.3-1.7)	2.3×10^{-8}

[a]Included psychiatric CNVs are found in supplementary table 2. [b]Test if CNV-carriers were more likely to participate in follow-up at age 18 compared to controls. [c]Test if subjects not participating in follow-up at age 18 had more NDPs (based on A-TAC) at age 9/12 compared to subjects participating (analyses within each CNV carrier group).

Supplementary Figure 1. CNV coordinates in human built hg19 for the included CNVs.

A. 15q11.2 (BP1-BP2) DEL/DUP.

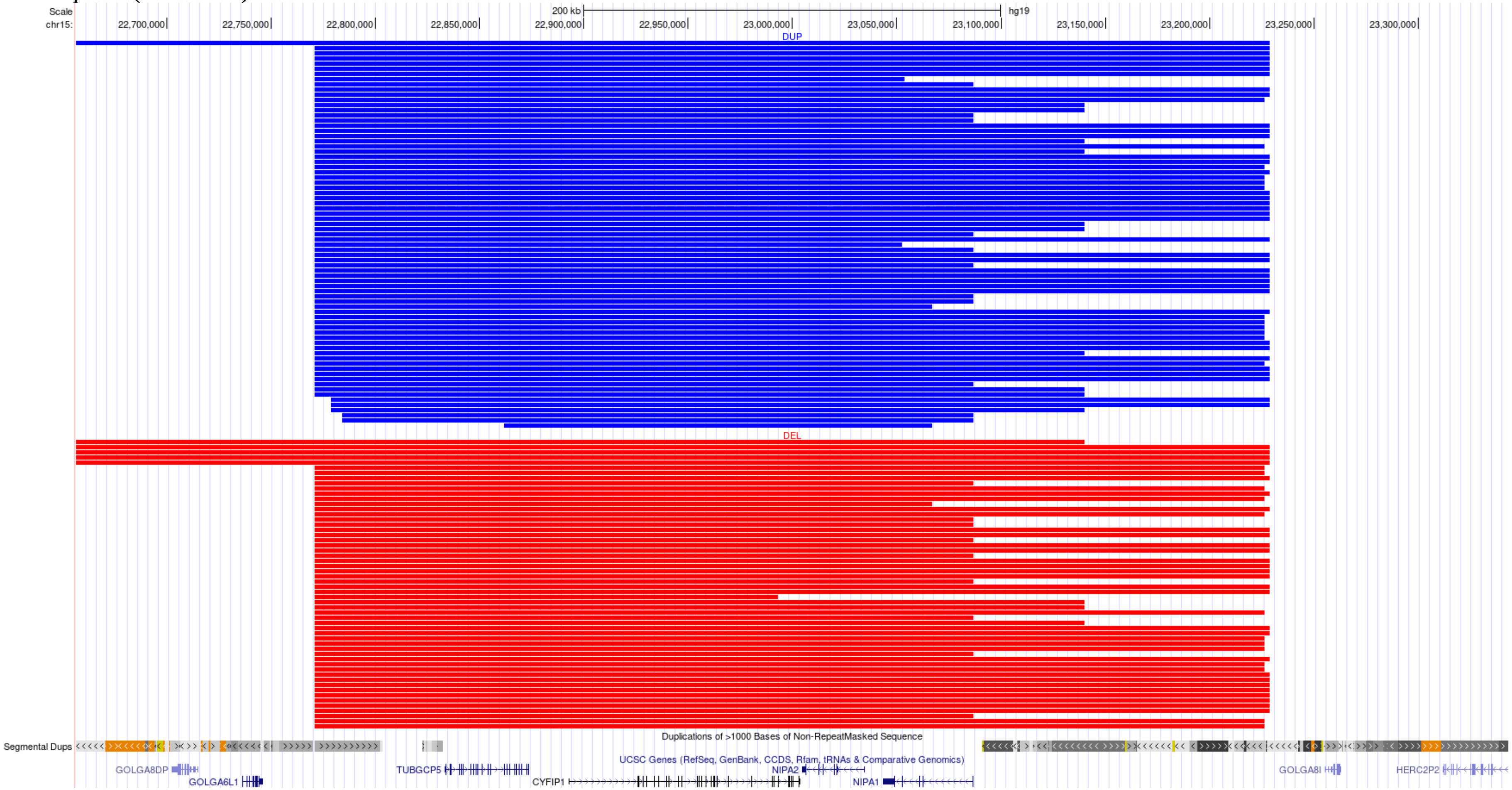

B. 1q21.1 distal_DEL/DUP

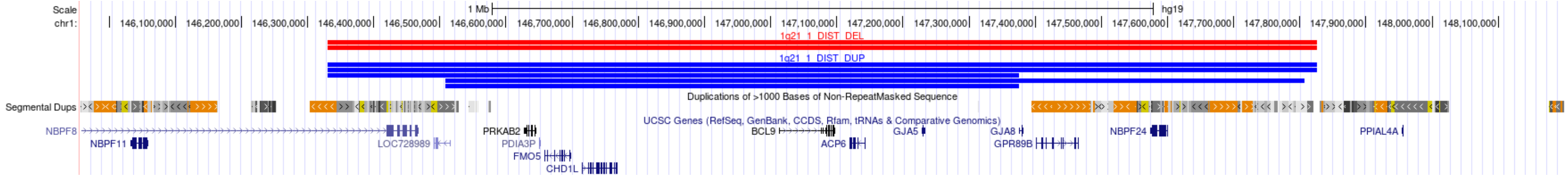

C. 2p16.3 (NRXN1) exonic DEL

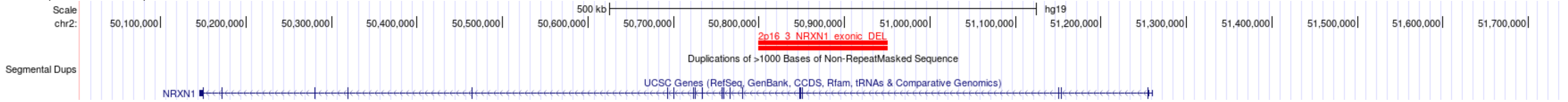

D. 15q11.2-13.1 DUP

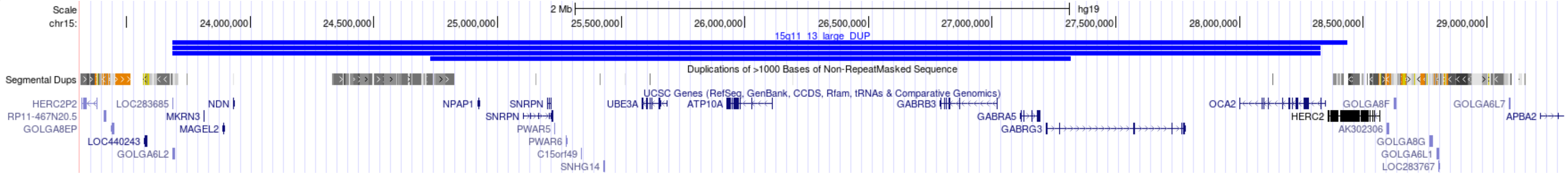

E. 15q13.3 DEL

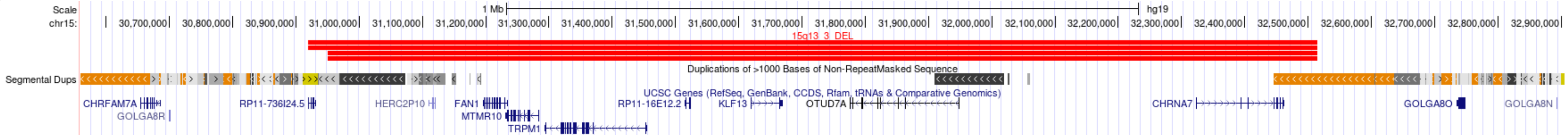

F. 16p11.2 distal DEL

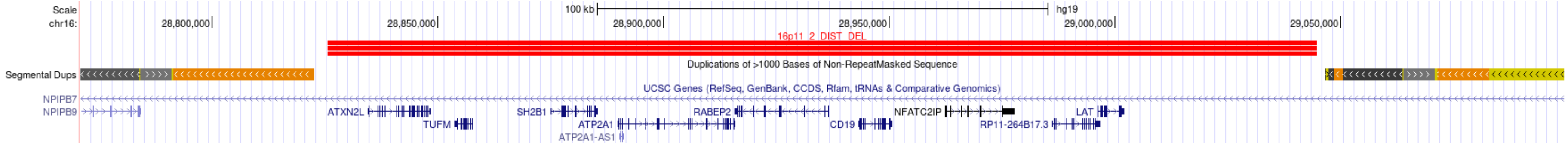

G. 16p11.2 proximal DEL/DUP

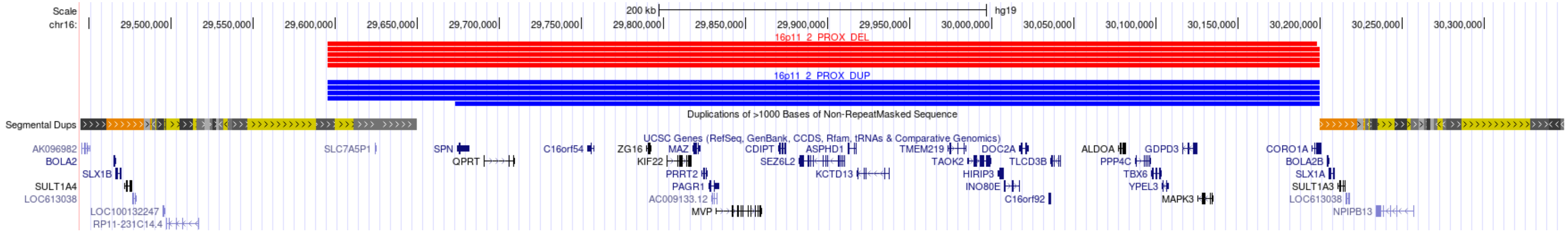

H. 16p13.11 DEL/DUP

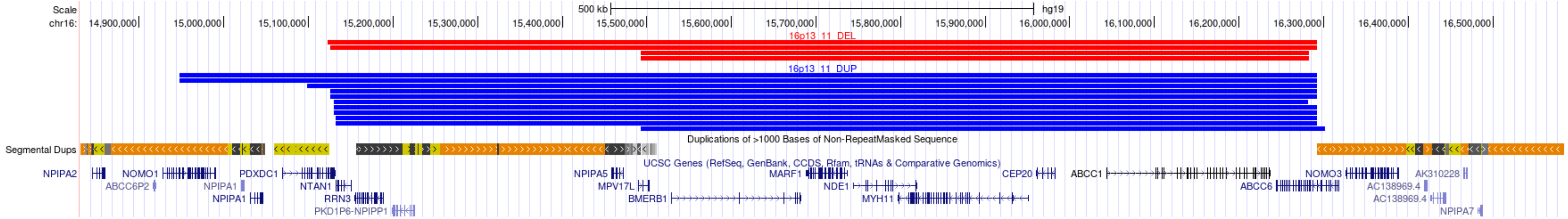

I. 17p12 DEL/DUP

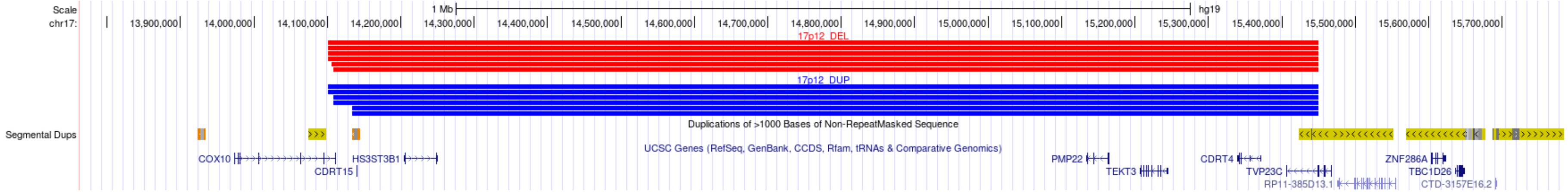

J. 22q11.2 DEL/DUP

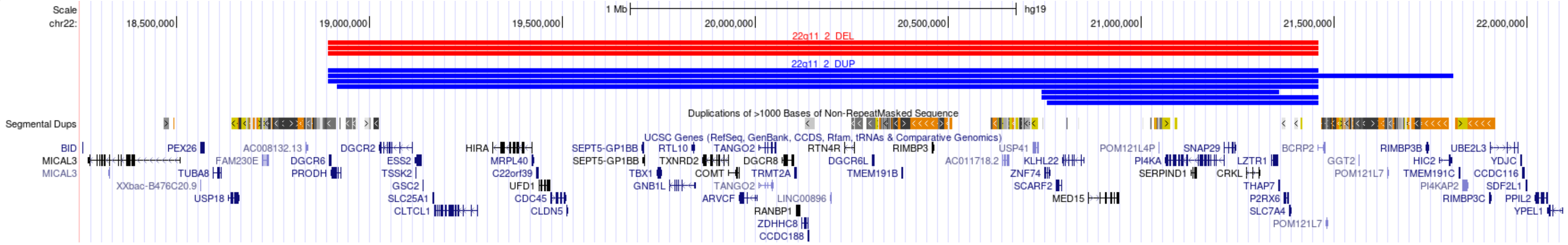

Supplement: Supplementary file 1 — Appendix S1. [file MGG3-11-e2191-s001.pdf]
